# Assessment of lipidomic species in hepatocyte lipid droplets from stressed mouse models

**DOI:** 10.1038/sdata.2014.51

**Published:** 2014-12-23

**Authors:** Jürgen Hartler, Harald C Köfeler, Martin Trötzmüller, Gerhard G Thallinger, Friedrich Spener

**Affiliations:** 1 Bioinformatics Group, Institute for Knowledge Discovery, Graz University of Technology, Petersgasse 14, 8010 Graz, Austria; 2 Omics Center Graz, Stiftingtalstr. 24, 8010 Graz, Austria; 3 Core Facility Mass Spectrometry, ZMF, Medical University of Graz, Stiftingtalstr. 24, 8010 Graz, Austria; 4 Department of Molecular Biology and Biochemistry, Medical University of Graz, Harrachgasse 21, 8010 Graz, Austria; 5 Department of Molecular Biosciences, University of Graz, Heinrichstr. 31, 8010 Graz, Austria

## Abstract

Lipid droplets are considered to be the hub for storage and metabolism of cellular lipids. In previous work we have phenotyped the lipidome of murine hepatocyte lipid droplets using liquid chromatography-mass spectrometry (UHPLC-MS) plus integrated MS/MS, followed by automatic analysis of the MS data. The organelles were isolated after intervention studies involving nutritional stress (extended feeding of a high fat diet or short term fasting), genetic stress due to knock-out of adipocyte triglyceride lipase, or by combined application of nutritional and genetic stress together (‘super stress’). Lipidomics at the level of lipid species (profiling of lipid classes) and lipid molecular species (structural analysis in parallel) has unraveled clear lipid droplet phenotypes as judged by patterns seen best in triacylglycerol (TG) lipidomes, but also in diacylglycerol and phosphatidylcholine lipidomes. The combined view of these data presented here validates the methods used and provides high quality lipidomic data for further bioinformatic inspections. Examples are given for identification of TG species subsets considered surrogates for whole TG lipidomes.

## Background & Summary

During the last ten years previously neglected lipid droplets (LDs) have come to the attention of researchers interested in cellular lipid metabolism^1^. This organelle has a neutral lipid core of mainly triacylglycerols (TG) and cholesterol esters, surrounded by a monolayer of mainly phospholipids (PL) and proteins^2–4^. LDs are not simply storage vessels for hydrophobic compounds, but are active functional units for metabolizing lipid molecules; they are involved in energy-linked metabolism, signaling, gene regulation, and autophagy^5–8^. Therefore, we have considered LDs as potential sensors for the physiological/metabolic state of the organism, a hypothesis that can be tested by a lipidomics approach^9,10^. Because liver is the central hub for lipid metabolism in health and disease^11–13^, we have analyzed hepatocyte LDs from mouse models that had been exposed to nutritional and/or genetic stress.

**Figure 1:**
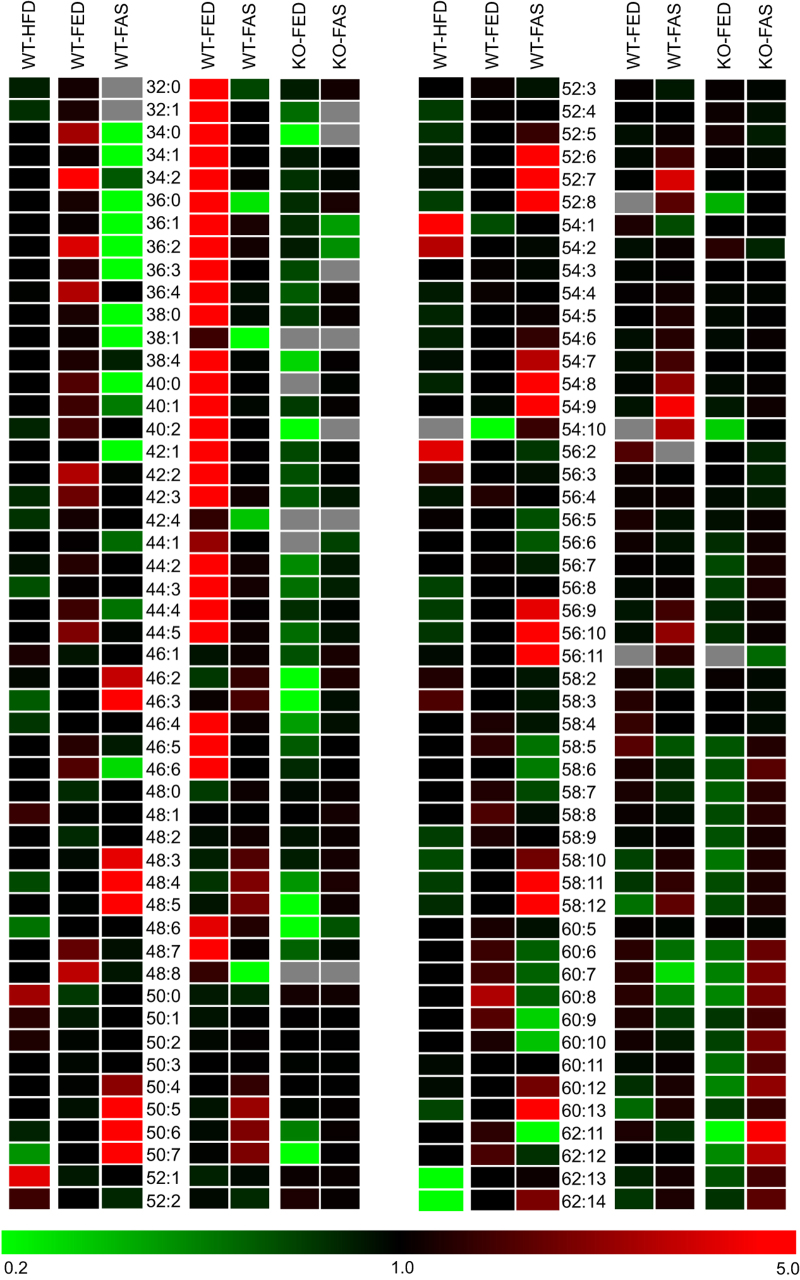
Heat map of TG species. Visualized are TG species detected by lipidomic analysis of hepatocyte lipid droplets in response to nutritional and genetic stress exerted to WT and ATGL-deficient mice. Left columns of species notation are sample groups pertaining to HFD-study (WT-HFD, WT-FED, WT-FAS; Data Citation 2), right columns to ATGL-study (WT-FED, WT-FAS, KO-FED, KO-FAS; Data Citation 3). TG class is best suited for phenotyping, illustrated here by red and green colors for increasing and decreasing species abundance relative to the mean of the 3 and 4 sample groups in the row for the HFD- and ATGL-study, respectively; the color tint indicates fold-changes up to five and down to 0.2. Black stands for no change, grey for not detected. In this way heat maps allow to single out marker species, such as TG 52:1, 54:1, 54:2 and 56:2 for high fat diet administration. Even minor compounds from TG 44:5 downwards, clearly visible in WT–FED sample groups in both studies, become drastically reduced in response to nutritional and genetic stress. Conversely, at the high end of the profile very minor TG species from TG 60:6 upwards become considerably enriched under super stress conditions (KO-FAS). The main issue is recognition of specific patterns, i.e., lipidomic phenotypes, for each stress condition applied to the animals. A case in point is from carbon numbers 48 to 58, where higher unsaturation becomes enriched in the HFD-study under fasting conditions, a phenomenon verified in the ATGL-study (WT-FAS). Data are based on means of MS-profiling of TG species in ‰ relative to total TG amounts with *n*=6 for HFD-study and *n*=3 for ATGL-study.

Our method of choice has been ultra-high performance liquid chromatography coupled to high resolution mass spectrometry (UHPLC-MS), capable of performing full scan MS parallel to low resolution collisional activated dissociation MS/MS. The pre-separation step had enabled us to overcome the challenge of bulk TG class present in LDs that otherwise would have blunted the MS-determination of a number of lipid species from PL classes. Simultaneously, we had developed algorithms for automatic high-throughput data evaluation, called Lipid Data Analyzer^14^. In a first method paper, we presented proof of principle for all methods elaborated^15^. Next, we designed intervention studies for nutritional stress with a wild-type strain fed lab chow (control), or exposed to either long-term high fat diet or to short-term fasting (‘HFD-study’)^16^. A further study addressed genetic stress by using an adipose triglyceride lipase (ATGL)-deficient strain fed either lab chow or being subjected to short-term fasting; in this case controls were again wild-type animals fed or fasted as before (‘ATGL-study’)^17^. At the end of the intervention periods livers were removed, hepatocyte LDs isolated, and total lipids extracted and subjected to lipidomic analyses by UHPLC-MS for profiling of *lipid species*. Such species are defined by their numbers of carbon atoms (chain-lengths): numbers of double bonds, not counting carbons of the glycerol backbone or head-groups, e.g., TG 52:3 or PC 36:4. Further structural elucidation was attained by MS/MS methodology, furnishing *lipid molecular species*. Pertinent structures are written as, e.g., TG 16:0_18:1_18:2 or PI 18:0_20:4, respectively^18^. Data obtained are summarized as follows:

About 98 mol% of total LD lipids belong to the TG class; remaining classes are, in quantitative order, DG, PC and other minor PL classes. Lipidomic analysis of lipid species resulted in distinct profiles, particularly for the TG class, but also for DG and PC classes. The TG lipidomes (over 100 species) ranged from TG 28:0 to 62:15, their presentation in heat maps indicated distinct patterns for each of the 3 groups in the HFD-study (nutritional stress) and 4 groups in the ATGL-study (genetic/nutritional stress). Subsequent principal component analysis (PCA) clearly separated in either case the 3 and 4 groups according to nutritional, genetic, or both stresses (‘super stress’). A third approach, calculation of average numbers of carbons and double bonds in acyl residues of total TG species, resulted in significantly distinct values for each physiological/metabolic state of the animals. Still clear enough were evaluations of the 26 DG species and 24 PC species detected in respective lipidomes. In summary, MS-profiling of lipid species and their heat map presentation identified marker species for HFD treatment and fasting of animals on the one hand (Figure 1), whereas these two modes of presentation, PCA separation and calculation of average numbers allowed for clear pattern recognition on the other hand. Further structural elucidation at the lipid molecular species level, resulting in more than one molecular species present in a defined lipid species, distinguished between different stresses applied and delineated metabolic relationships between lipid molecular species of different classes^16,17^.

**Figure 2:**
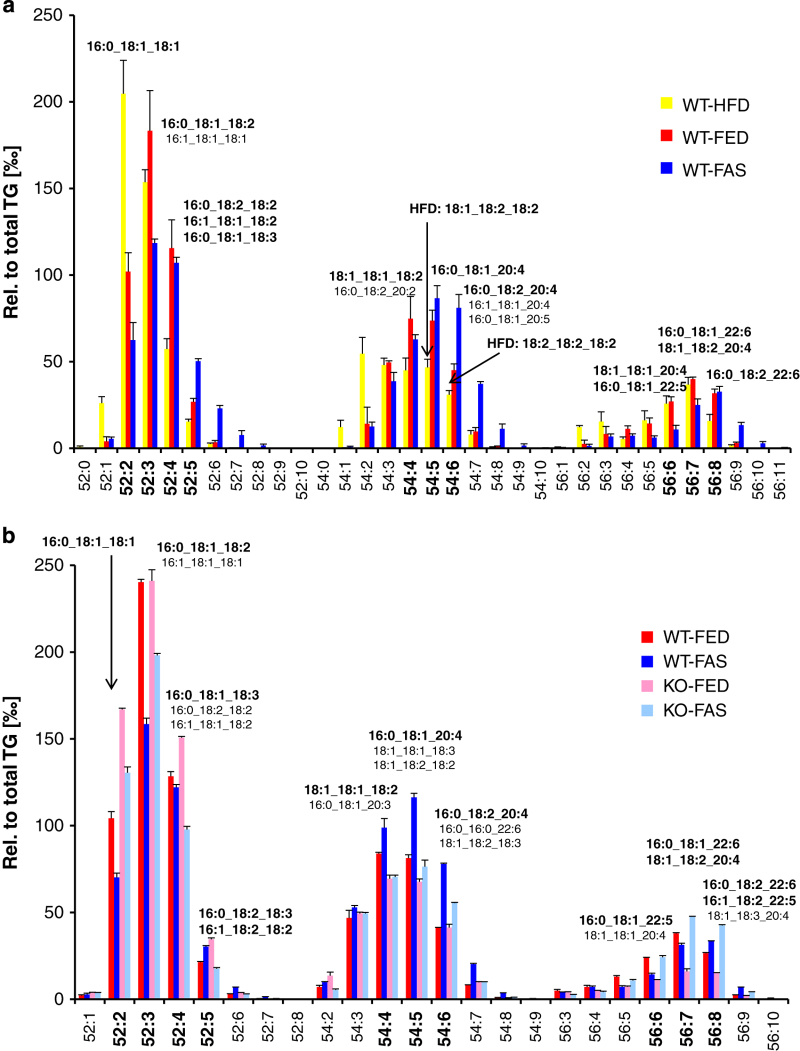
Detection of lipid molecular species. Shown here are profiles of TG species and TG molecular species with carbon numbers from 52–56, determined by UHPLC-MS and MS/MS, respectively, in ‰ relative to total TG amounts. In the HFD-study (Data Citation 2) (**a**) bars represent means±s.d. (*n*=6) and in the ATGL-study (Data Citation 3) (**b**) means±s.d. (*n*=3). All TG species enumerated at the abscissa form profiles phenotypic for the stresses applied, TG species in bold are some discriminants for separation of sample groups by PCA (Figure 4b). Structures of isobaric TG molecular species, result from different assemblies of constituent fatty acids esterified to glycerol and are written above the bars for ‘bold’ TG species. Here too, predominant molecular species are found (shown in bold as well). It is noteworthy that very long-chain fatty acids (FA 20:4 and 22:6) are found in TGs from carbon numbers 54 on. For a given lipid species, composition of lipid molecular species can vary from sample group to sample group, a further criterion for phenotyping as we reported earlier^16^.

**Figure 3:**
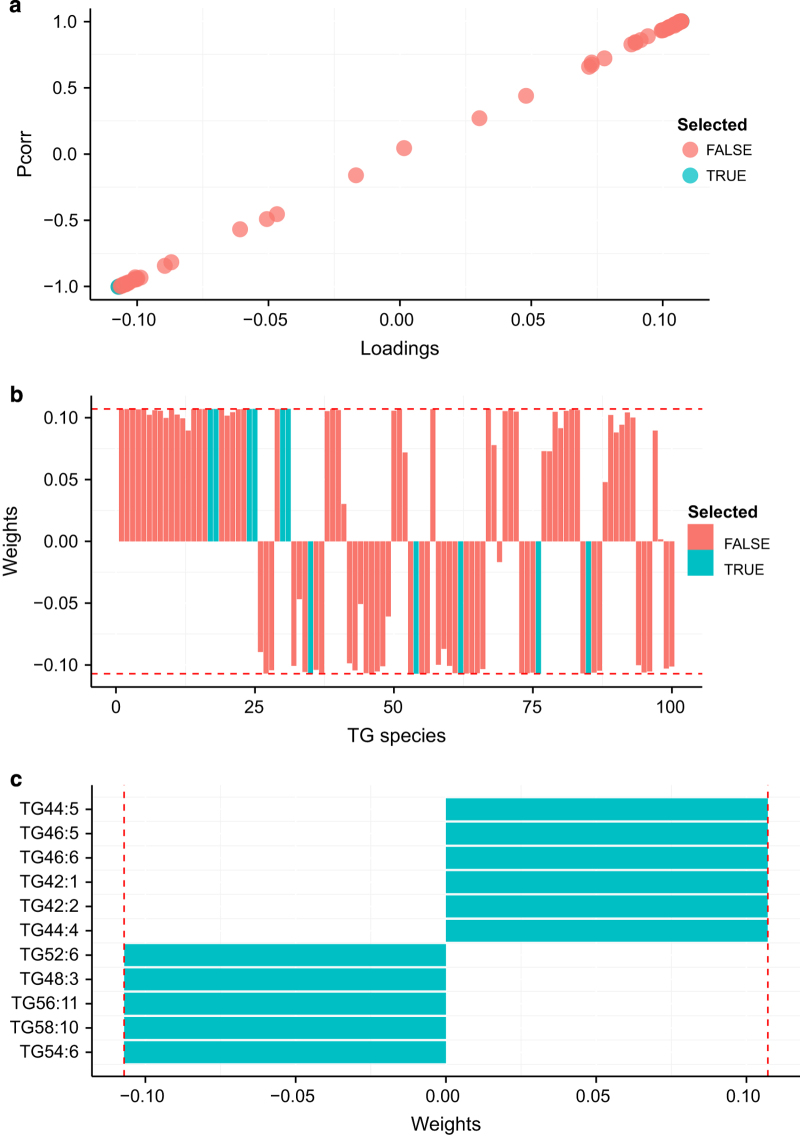
Discriminatory TG species identified by O-PLS-DA. Species as identified for separation of WT-FED from WT-FAS sample group in the ATGL-dataset (Data Citation 3) are colored in light blue. (**a**) The S-plot over all TG-species shows the loading of a TG species versus its correlation to the score. (**b**) Weighted loading plot of all TG species. (**c**) Plot of discriminatory TG species and their weighted loadings.

**Figure 4:**
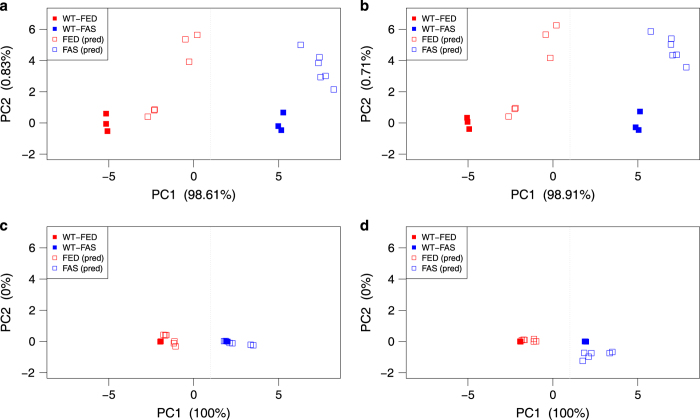
Verification of discriminatory TG species on independent HFD-dataset. Separation and classification of TG species from WT-FED and WT-FAS sample groups taken from HFD-data (Data Citation 2) based on PCAs from respective ATGL-data (Data Citation 3). Scatter plots were generated using the TG lipidome (**a**), and subsets of TG species obtained by PCA analysis (**b**), species from O-PLS-DA showing highest positive and highest negative correlation (**c**), and species from O-PLS-DA showing highest negative correlation (**d**). Filled rectangles indicate data from the training set (ATGL, *n*=3), and empty rectangles correspond to data from the independent validation set (HFD, *n*=6). All approaches show a clear separation of the independent validation data, and all samples are projected in close distance to the correct group of the ATGL training set. Surprisingly, the smallest subset (**d**) classified the independent validation data closest to the training data. pred, prediction for classification of independent validation data.

Based on these previous observations, we have now revisited our data for streamlining and validation of the two studies, and to assist other researchers in using these data. We offer approaches for screening TG-lipidomes for some discriminatory lipid species (written in bold in abscissas in Figure 2) that phenotype the physiological/metabolic state of the animal model. PCA and orthogonal partial least square discriminant analysis (O-PLS-DA) approaches (Figures 3 and 4), and average calculations (Table 1), enable identification of lipid species subsets as surrogates for whole lipidomes.

## Methods

Descriptions are taken from our previous work^14–17^ either completely, or adapted, or supplemented with new details where necessary. Additional tables and figures, labeled with an 'f' prefix, can be found at figshare (Data Citation 1).

### Biological work

#### Materials

Collagenase type II was purchased from Worthington Biochemical Corp. (Lakewood, NJ, USA) and Narkodorm from CP-Pharma (Burgdorf, Germany). Cell strainers were obtained from Becton Dickinson GmbH (Heidelberg, Germany). A nitrogen bomb was purchased from Parr Instrument Comp. (Moline, IL, USA). Polyvinylidene fluoride membrane was obtained from Pall Life Sciences (Pensacola, FL, USA). Antibodies against perilipin 2 (ADRP) were obtained from Progen Biotechnik (Heidelberg, Germany), all other antibodies from Cell Signaling Technology, Inc. (Danvers, MA, USA). ECL plus and Hyperfilm ECL were from GE Healthcare Europe GmbH (Vienna, Austria). All other chemicals were purchased either from Merck KGaA (Darmstadt, Germany), Sigma-Aldrich (St Louis, MO, USA) or Roth GmbH & Co.KG. (Karlsruhe, Germany). Lipid standards were obtained from Avanti Polar Lipids Inc. (Alabaster, AL, USA).

#### Animals, diets and intervention studies

All animal experiments were performed in compliance with the Austrian animal protection law. The mice were housed and handled in accordance with good animal practice as defined by FELASA (www.felasa.eu/guidelines.php). The animal welfare committees of the University of Graz and the national authorities approved all animal experiments. Male wild-type (WT) and male ATGL-nullizygous C57BL/6 mice (KO)^19^ were used, aged six weeks at the start of the studies. Mice were maintained on a regular light (14 h)—dark (10 h) cycle and fed lab chow for six weeks (FED groups), or maintained on high fat diet (HFD groups), or were fasted 14 h prior to sacrifice (FAS groups). All diets were from sniff® Spezialdiäten (Soest, Germany) and are shown in Tab. f1 (Data Citation 1). Three animals per group were kept in one cage with *ad libitum* access to food and water. During the intervention period mice weights were monitored regularly. At the end of the six weeks trial periods, animals were euthanized at 8 AM under anesthesia. The HFD-study was run consecutively twice with always 3 mice per group (WT-HFD, WT-FED, WT-FAS)^16^. Due to excellent agreement of data they are combined and are reported as *n*=6. One year later, therefore, we carried out the ATGL-study with 3 mice per group (WT-FED, WT-FAS, KO-FED, KO-FAS) only once (*n*=3)^17^. For the sake of clarity, animals and diets used in the various sample groups are summarized here:

WT-FED: wild-type mice fed lab chow;

WT-FAS: wild-type mice fed lab chow, but fasted 14 h prior to sacrifice;

WT-HFD: wild-type mice fed high fat diet;

KO-FED: ATGL-knock out mice fed lab chow;

KO-FAS: ATGL-knock out mice fed lab chow, but fasted 14 h prior to sacrifice.

#### Isolation of hepatocytes

Mice were anesthetized subcutaneously with Narkodorm (60 μl/100 g weight) and the abdomen was surgically opened. Primary hepatocytes from livers were isolated according to Riccalton-Banks *et al.* with modifications^20^. Prior to liver perfusion, buffers and collagenase type II solution (20 mg collagenase type II in 100 ml Krebs-Henseleit buffer without SO_4_^2−^ but containing 0.1 mM CaCl_2_, 2% bovine serum albumin) were brought to 37 °C. Each liver was perfused *via* hepatic portal vein with Krebs-Henseleit buffer without Ca^2+^ and SO_4_^2−^ (115 mM NaCl, 25 mM NaHCO_3_, 5.9 mM KCl, 1.18 mM MgCl_2_, 1.23 mM NaH_2_PO_4_, 6 mM glucose) for 10 min, followed by perfusion with collagenase type II solution. Thereafter, each liver was carefully removed, transferred into a Petri dish, filled with 5 ml collagenase type II solution, cut into small pieces, pressed through a household sieve which was finally flushed with ice-cold Krebs-Henseleit buffer containing 1.2 mM Na_2_SO_4_ and 1.25 mM CaCl_2_. The cell suspension obtained was filtered through a cell strainer (70 μm nylon filter) into a 50 ml Greiner-tube. Approximately 20 ml ice-cold Dulbecco’s Modified Eagle's Medium (DMEM) was added to the filtered cell suspension, which was subsequently centrifuged at 50 g in a Beckman CS-6R rotor for 3 min at 4 °C. Supernatant, containing non-parenchymal cells, was aspirated and the remaining hepatocyte pellet was washed by re-suspension of the cell pellet in 20 ml ice-cold Krebs-Henseleit buffer containing 1.2 mM Na_2_SO_4_ and 1.25 mM CaCl_2_ and centrifuged again under conditions as applied before. Hepatocytes obtained in this manner were stored at −80 °C until isolation of LD.

#### LD isolation from hepatocytes by nitrogen cavitation

Samples of hepatocytes isolated from individual mice were re-suspended in disruption buffer (20 mM potassium phosphate pH 7.4, 250 mM sucrose, 1 mM EDTA, 1 mM PMSF) and kept for 15 min on ice. The cells were lysed by nitrogen cavitation at 800 psi for 10 min^21^ using a nitrogen bomb and the resulting homogenate was transferred to a 50 ml Greiner-tube and centrifuged at 1,000 g (Beckman CS-6R rotor) for 5 min at 4 °C to remove cell debris. The supernatant obtained was transferred to an SW41 ultracentrifuge tube and overlaid with buffer containing 50 mM potassium phosphate pH 7.4, 100 mM KCl, 1 mM EDTA, 1 mM PMSF. The centrifugation was carried out at 100,000 g in a Beckman ultracentrifuge (SW41 rotor, Beckman Coulter, Brea, CA, USA) for 1 h at 4 °C; LDs floated in a white band at the top of the tube. Floating LDs were collected into another SW41 tube and overlaid with the same buffer and centrifuged at the same speed in the Beckman ultracentrifuge (SW41 rotor) for 1 h at 4 °C, in order to avoid cytosol contamination. Re-floating LDs were collected and used in all further experiments.

### Sample preparation for LC-MS analysis

Lipid droplets isolated from hepatocytes were measured in individual samples per group. Lipid extraction was carried out by a methyl *tert*-butyl ether (MTBE) protocol as described previously^22^. In brief, each sample of the HFD-study was spiked with 40 nmol TG 17:0/17:0/17:0 and 400 pmol PC 12:0/12:0 as internal standards, the samples of the ATGL-study were spiked with 40 nmol TG 17:0/17:0/17:0, 1.2 nmol PC 12:0/12:0, 2.4 nmol PE 12:0/12:0, and 4 nmol PS 12:0/12:0 as internal standards. This set of internal standards was used to ascertain constant extraction efficiency for all samples in a batch. Briefly, for each internal standard the mean of all samples in a batch was calculated and any sample with an internal standard deviation of more than 30% was rejected. Subsequently, a volume of 3 ml methanol and then 10 ml MTBE were added to 2 ml of LD suspension and tubes were shaken for 1 h at room temperature. Upon addition of 2.5 ml deionized water and shaking, phase separation was induced. The upper organic phase was collected, the lower aqueous phase re-extracted with MTBE and upper phases were combined. The solvent was removed *in vacuo* (SpeedVac, Thermo Fisher Scientific, San Jose, CA, USA) and lipid extracts were re-suspended in 200 μl CHCl_3_/MeOH 1:1. Thereafter a mix of 45 LIPID MAPS internal standards (Tab. f2, Data Citation 1) was added for internal calibration and subsequent calculations.

### UHPLC-MS analysis

The UHPLC system (Accela, Thermo Fisher Scientific, Bremen, Germany) was equipped with a reversed-phase C18 column (100×1 mm i.d., 1.9 μm particle size). Mobile phase A was 10 mM ammonium acetate containing 0.1% formic acid. Mobile phase B was acetonitrile/2-propanol 5:2 (v/v) containing 10 mM ammonium acetate and 0.1% formic acid. The binary gradient started with 35 to 70% B in A for 4 min, then was raised up to 100% B in another 16 min and further held for 10 min. The flow rate was 250 μl/min, the oven temperature was 50 °C and tray temperature 4 °C. For analysis 5 μl sample were injected. A 7.0 Tesla LTQ-FT hybrid linear ion trap Fourier transform ion cyclotron resonance mass spectrometer (Thermo Fisher Scientific, Bremen, Germany), equipped with an electrospray ion source, was used for mass spectrometric determination. The instrument was operated in data dependent acquisition (DDA) mode on the 4 most abundant precursor ions for parallel MS/MS spectra in the linear ion trap, while running the ion cyclotron in full scan mode at 200,000 resolution (m/z 400) from m/z 350 to 1,050 in positive and from m/z 200 to 1,500 in negative ESI-mode. Helium was used as damping gas for linear ion trap collision-induced dissociation (CID) spectra. The following parameters were used for positive and negative DDA MS/MS experiments: Normalized collision energy was 35%, the repeat count was 2 and the exclusion duration 60 s. The activation Q was at 0.2 and the isolation width 2. For positive ESI spray voltage was set to 5 kV and the tube lens offset was at 120 V. For negative ESI spray voltage was – 4.8 kV and the tube lens offset was – 87 V. The sheath gas flow was set to 50 arbitrary units, auxiliary gas flow to 20 arbitrary units, sweep gas flow to 2 arbitrary units and the capillary temperature to 250 °C^15^.

### Data processing

Identification and quantitation of lipid species was performed using LDA, a stand-alone Java application developed by us^14^ (available from http://genome.tugraz.at/lda). Briefly, the algorithm identifies peaks in the three dimensional LC-MS data space (retention time, m/z and intensity), determines peak borders in m/z as well as in time dimension, and integrates the intensities within the borders. Furthermore, the algorithm uses the theoretical isotopic intensity distribution as peak selection criterion to improve specificity. Data were normalized to the total amount of a lipid class or whole lipidome. For class specific normalization, no standardization is required, since similar ionization efficiencies within a lipid class, e.g., TG, can be assumed. Thus, the shown values in ‰ (or %) are the signal contributions of each species in relation to the total class signal detected by MS. In contrast, for lipidome normalization, data were first normalized on multiple internal standards as described (Chapter 3.3 in ref. 14, robust standardization), in order to infer the molar amounts of lipid species. Second, data were normalized to the sum of these molar contributions to obtain comparable relative contributions of each lipid species. Quantitative data presented are expressed in ‰ (or %) relative to total amount of a lipid class, or total amount of lipidome, respectively. We favored data presentation relative to total amounts to compensate for potential differences in extraction. Shorthand notation for MS-lipid data used here was developed by us and is based on LIPID MAPS nomenclature and abbreviations^18^.

LDA identifies lipids based on the number of carbon atoms and double bonds. Thus, constituent fatty acids (FA) esterified in TG species were determined in positive MS/MS spectra by respective FA neutral losses. The most abundant fragment^23^ in each MS/MS spectrum is normalized to 100% intensity (base peak). Fatty acyl fragments or FA neutral loss fragments with less than 25% intensity of base peak are termed minor, all other major FA fragments. For better manageability, particularly of isobaric species, possible combinations predominantly composed of major fragments are addressed major molecular species, those predominantly composed of minor fragments minor molecular species.

### Statistical analysis of MS data

Quantitative data for each lipid species are expressed in ‰ (or %) relative to total amount of the respective lipid class and pertain to HFD-study (means±s.d. of 6 animals, Tab. f3 in Data Citation 1, Figure 2a) and ATGL-study (means of 3 animals, Tab. f4 in Data Citation 1, Figure 2b). Profiles for other lipid classes from both studies can be found in Fig. f5 (Data Citation 1). The choice of relative values enabled measuring compositional changes in respective lipidomes in a quantitative manner. Independent two-sample *t*-test for equal sample sizes and equal variance was used to assess statistical significance of the observed changes at levels of *P*<0.05, 0.01, and 0.001, respectively.

### Statistical analysis by PCA

Lipid species data from the ATGL-study in ‰ relative to total amount of a lipid class or whole lipidome (Tab. f4), respectively, are further analyzed by PCA. This is performed in R^24^ (version 3.1.0) using the function prcomp with default parameters. The loadings of the TG species calculated in PCA, stored in Tab. f6 (contains data for DG and PC classes as well, Data Citation 1), led to clear separation of TG species between sample groups of mice under the various stresses applied to the animals (Figure 2a in ref. 17 and in Fig. f7 of Data Citation 1). Based on these results, we can now detect lipid species whose abundance levels are significant for a stressed state. Since PCA analysis is in fact a coordinate transformation, where each species measurement contributes additively, the contribution of each species is measurable. Consequently, a transformed principal component (PC) coordinate (=factor score) can be illustrated in scatter plots (e.g., Figure 4) and is calculated as follows:

(1)PCx=∑i=1nmi*lx,i

*m*_*i*_ corresponds to the measured abundance of lipid species *i*, *x* is the index of the principal component (e.g., PC1, PC2, etc.), *l*_*x,i*_ represents the loading for lipid species *i* in principal component *x* and *n* denotes the number of lipid species. In order to detect species contributing the most to the discriminatory effect between sample groups, we calculated the mean of each lipid species for each sample group, subtracted the means from one another, and multiplied it by the loading, e.g.,:

ΔPC1,WT−FAS−KO−FAS(TG52:2)=(meanWT−FAS(TG52:2)–meanKO−FAS(TG52:2))*(2)l1,iΔPC1,WT−FAS−KO−FAS(TG52:2)=(70.01–130.40)–0.6189=37.38

The mean of sample group with the lower PC1 value was subtracted from the one with the higher one. Consequently, for PC1, positive values strengthen the evidence of difference between sample groups, negative values argue against. The same was done for PC2, but sample groups with a more positive value in PC1 may be more negative in PC2, in such case the negative values may be the ones that strengthen the evidence of a difference. In order to avoid confusion, we place species supporting separation at the top and contradicting and minor contributions at the bottom of ‘Contribution tables’ stored in Tab. f8 and Tab. f9, both in Data Citation 1.

This shows clearly that some species contribute to the separation of the individual groups of stressed animals to a much higher extent. Consequently, we presume that a low number of lipid species is sufficient to phenotype the physiological/metabolic state of the animal. The major contributors of TG class were TG 52:2, 52:3, 52:4, 52:5, 54:4, 54:5, 54:6, 56:6, 56:7, and 56:8 (10 species).

### Statistical analysis by O-PLS-DA

This multivariate data analysis^25^ method aims to transform the data into a new coordinate system, where the variance between groups is maximized in the first transformed dimension. Lipid species data from the ATGL-study in ‰ relative to total amount of a lipid class or whole lipidome (Tab. f4), respectively, were analyzed in R^24^ (version 3.1.0) using the *DEVIUM* platform for multivariate analysis (http://github.com/dgrapov/devium). Lipid species contributing the most to the discrimination between sample groups were identified based on their correlation to the scores and their weighted loadings. This relationship can be visualized in form of an S-plot (Figure 3a). The following TG species were putative candidates for discriminating WT-FED from WT-FAS sample groups: TG 42:1, 42:2, 44:4, 44:5, 46:5, and 46:6 showing a positive correlation, and TG 48:3, 52:6, 54:6, 56:11 and 58:10 with a negative correlation, respectively (Figure 3b,c). We analyzed both subsets and their union by PCA (Fig. f10, Data Citation 1). All three subsets separated the WT-FED and the WT-FAS data well, but the absolute distance between the groups in PC1 was much higher by the negative subset, and the loadings of the union subset were dominated by elements of the negative subset. Consequently, we used as subsets of the O-PLS-DA results only the species showing negative correlation (5 species) and the union of positive and negative correlation (11 species) for subsequent analysis.

### Verification of discriminatory species identified by PCA and O-PLS-DA

To verify the capability of separating phenotypes by subsets of lipid species, selected by statistical methods, we tested the classification of sample groups from the independent HFD-dataset^16^ based on PCA loadings derived from our TG subsets of the ATGL-dataset (training data)^17^. Classification was performed using the R function predict with default parameters. Since the HFD-study is based on the wild-type genotype only, the classification is based on sample groups WT-FED and WT-FAS of the ATGL-study.

We tested the predictive ability for phenotyping the animals’ physiological/metabolic states on the whole TG lipidome (100 species, Figure 4a), a set of species identified by PCA (10 species, Figure 4b), and two subsets identified by O-PLS-DA (11 and 5 species, Figure 4c,d, respectively). All approaches show a clear separation of the independent validation data, and all samples are projected in close distance to the correct group of the ATGL training set. Surprisingly, the smallest subset, the negative O-PLS-DA where 100% of the discriminative power is in PC1, grouped the independent validation data closest to the training data (Figure 4) and performs as such much better than a classification based on the whole TG lipidome.

### Statistical analysis by averaging numbers of carbons and double bonds of main lipid species

A rather simple approach to gauge phenotypes of lipid species profiles is averaging chain-lengths of constituent fatty acids (=the numbers of carbon atoms) and the numbers of double bonds contained therein. On the one hand, due to the relationship between chain-elongation and desaturation during fatty acid biosynthesis or degradation the number of double bonds always increases or decreases with the number of carbon atoms. On the other hand, the lipid profile is based on abundances of each lipid species within the lipidome, consequently the most abundant species will contribute more to average values than less abundant ones. We have shown these relationships earlier (Figure 3 in ref. 17) for the whole lipidomes of TG, DG, and PC classes, respectively. Here we average TG species only. Since four physiological/metabolic states are investigated in the ATGL-study, the mean of the areas of all four sample groups are taken and the reference is the peak for the lipid species of highest abundance per class (base peak=100%). When the selection level was set to 25% and above relative to base peak, six TG species were singled out and used for averaging. The values obtained and presented in Tab. 1 are lower than those based on whole TG lipidome, due to overall less abundance. Importantly, significant differences characteristic for the phenotypes are as clear as seen for the whole TG lipidome. When the selection value was set to e.g., 10%, 11 TG species were identified and subsequent averaging furnished similar results.

## Data Records

### Data record 1

Detailed data are uploaded as files in figshare, indicated by an ‘f’ (Data Citation 1).

**File 1: Tab. f1—Animal diets**: Normal chow (control) and high fat diet.

**File 2: Tab. f2—List of internal standards**: Internal lipid standards used in HFD- and ATGL-studies for extraction control, for calibration in UHPLC-MS and for quantitative calculation of lipid species.

**File 3: Tab. f3—Lipidomes from HFD-study**: Quantitative lipid species data and structures of respective molecular species obtained by LC-MS and MS/MS analysis in HFD-study.

**File 4: Tab. f4—Lipidomes from ATGL-study**: Quantitative lipid species data were obtained by LC-MS analysis. The values correspond to the relative contributions of each lipid species to the whole lipidome, TG-, DG-, and PC lipidomes, respectively. Mean values shown here were used for PCA analysis (e.g., Fig. f7).

**File 5: Fig. f5—Selected PL and DG species from HFD- and ATGL-studies**: Profiles of main species determined for DG, PC, PE, PS, and PI classes by UHPLC-MS in ‰ relative to total amount of respective class.

**File 6: Tab. f6—PCA loadings of the four lipidomes from ATGL-study:** The columns indicate the principal component affected and whether the contribution is positive or negative (e.g., PC1- contributes to a more negative outcome). The rows correspond to individual lipid species that are sorted by the absolute value of their loadings.

**File 7: Fig. f7—PCA** scatter plot for whole lipid droplet lipidome from ATGL-study: The plot demonstrates clear separation of the 4 sample groups.

**File 8: Tab. f8—PCA contributions of whole lipid droplet lipidome from ATGL-study:** The columns indicate the affected principal component. The rows contain the lipid species sorted by their discriminative contribution to the separation to the sample groups. Species supporting separation are at the top; contradicting and minor contributions are at the bottom of the tables. In this analysis, we investigated each species for its discriminatory effect between the sample groups. The same applies to file 9.

**File 9: Tab. f9—PCA contributions of lipid droplet lipidome from ATGL-study.**

**File 10: Fig. f10—PCA scatter plot of most discriminatory TG species identified by O-PLS-DA in ATGL-data:** TG species are candidates for discriminating WT-FED from WT-FAS sample groups. Shown are PCA scatter plots of TG species with positive correlation, negative correlation, and the union of both, respectively.

**File 11: Fig. f11—Validation of linearity**: Determination of the equimolar TG standard mix LM 6000 by LC-LTQ-FT reveal only minor differences in ionization efficiency and a high degree of linearity between 40 amol and 656 fmol absolute amount injected on HPLC column.

### Data record 2

#### Lipidomic raw data from MS

These data are deposited at the MetaboLights database of the European Bioinformatics Institute (EBI), (http://www.ebi.ac.uk/metabolights), the identifier for the HFD-study is MTBLS26 (Data Citation 2), for the ATGL-study MTBLS81 (Data Citation 3).

## Technical Validation

### Validity of the LC-MS/MS method

Technical validation of the LC-MS/MS method was attained by non-endogenous lipid standards spiked at various concentrations into an LD sample. This was carried out before we started sample measurement and was described in more detail earlier^15^. The reference standards used in this procedure are TG 17:0/17:0/17:0, PC 12:0/12:0, PE 12:0/12:0 and PS 12:0/12:0. In a nutshell, reference standards are quantified by our LDA algorithm as described in the data processing section by a one point calibration. The reference point here is the median of all internal standards of a lipid class distributed over the whole retention time range of respective lipid class. The number of internal standards varies from 10 to 4 per lipid class (Tab. f2). LC-MS/MS method validation shows accuracy values from 85.8 to 116.1% at medium concentration range of the calibration curve. Method precision for five technical replicates including sample preparation is between 4 and 9.6%. This is well in accordance with Food and Drug Administration (FDA) guidelines for targeted LC-MS methods which propose a maximum of 15% CV. Although PC 12:0/12:0 and PS 12:0/12:0 are slightly above the proposed 15% deviation for concentration accuracy, one has to consider that our method is not a targeted single reaction monitoring (SRM) assay usually used for quantitation. Rather it is a non-targeted omics approach where higher deviations are to be expected due to the nature of the LC-MS set-up. The dynamic range of the method covers more than 4 orders of magnitude and is between 40 amol and 656 fmol on column. Linear fits for calibration curves in this concentration range yield correlation coefficients between 0.952 and 0.981, which again is acceptable for a non-targeted omics method. Furthermore, comparability of internal standards across the whole retention time range by equimolar mixtures of 8 internal TG standards at 5 different concentration points is shown in Fig. f11 (Data Citation 1). Quantitative precision of these 8 compounds is 9%, which is well within the expected and tolerable range and allows reliable quantitation. Additionally, the relationship of the different concentration points of internal standards is highly linear, thus allowing comparison of measurements with different internal standard concentrations.

### Overall validity of the assay

Here we checked the validity of the data in a comparison of the two separately derived data sets from the two trials within the HFD-study. Of course, this kind of comparison also factors in the biological variance, not only the technical variance. It is thus considered to be an appropriate tool for the validity of our overall results, in a sense the whole pipeline from mouse to data analysis. Normalization of lipid species is given by using their ‰ values relative to total molar amount for each lipid class in respective samples, as evidenced above by using such data in the verification process across HFD- and ATGL-studies. The comparison of the lump sums of all lipid species from all lipid classes investigated resulted in an average deviation of per mille values to 25.9% between the HFD-trials. Application of the same procedure to comparison of the overall HFD-study with the ATGL-study, the deviation rose to 36.6%. If only main lipid species, which make up 90% of the LD-lipidome are taken into calculation, the deviation between HFD- and ATGL-studies decreased impressively to 19.0%. This reflects the fact that lipid species with higher abundance have much higher signal intensities and thus an increased quantitative confidence compared to minor species detected close to the limit of detection (Fig. f5).

## Usage Notes

The lipidomic data acquired by the Thermo LTQ-FT instrument are publicly available in ‘.raw’ file format at European Bioinformatics Institute’s MetaboLights repository (http://www.ebi.ac.uk/metabolights). After download the data are available for analysis by different software packages. In fact, this repository contains raw data of the whole lipidomes determined in lipid droplets. They enable application of bioinformatic processing, as shown in this paper for TG-lipidomes, to all other lipid classes. The data reported here already demonstrate the potential to diagnose hepatocyte lipid droplets obtained from diet- or genetically induced steatotic livers. Beyond a diagnostic value in general, such data provide insights into metabolic paths and how to interfere in them. Further files illustrate in detail the data necessary to execute PCA and O-PLS-DA as general methods for phenotyping. Finally, targeted and quantitative data obtained by LC-MS technology can be automatically processed and rapid quantitative evaluation of data can now easily be carried out by LDA (http://genome.tugraz.at/lda). Another software option for quantitative analysis is Lipid Search (Thermo Scientific, San Jose, CA, USA). Non-targeted differential lipidomic profiles featuring global differences between the sample groups can be obtained by Sieve (Thermo Scientific, San Jose, CA, USA).

The data sets documented here will allow singling out small subsets of lipid species indicative for a physiological/metabolic state. Such classification abilities can be validated on an independent data set. In fact, subsets of lipid species are sufficient to describe the state of an animal, and as such they can be regarded as true surrogates for extended lipidomes and even as potential candidates for biomarkers.

## Additional information

**How to cite this article:** Hartler, J. *et al.* Assessment of lipidomic species in hepatocyte lipid droplets from stressed mouse models. *Sci. Data* 1:140051 doi: 10.1038/sdata.2014.51 (2014).

## Supplementary Material



## Figures and Tables

**Table 1:** Phenotyping structural features in selected TG species.

	**WT-FED**	**WT-FAS**	**KO-FED**	**KO-FAS**
TG cl	52.608±0.006	52.910±0.010* ^,2^	52.484±0.008* ^2,^ † ^,2^	52.644±0.014*^1^, † ^2,^ ‡ ^,2^
TG db	3.581±0.013	3.959±0.007* ^,2^	3.424±0.011* ^2,^ † ^,2^	3.568±0.015† ^2,^ ‡ ^,2^
Nutritional and genetic stresses can be calculated by average number of carbons and average number of double bonds in acyl residues of selected species from the TG lipidome. Data are means±s.d. (*n*=3) and are based on the sum of selected species abundances in ‰ relative to total amount of TG class. Selection criterion is 25% or more relative to the most abundant TG species (=100%). cl=chain-lengths (number of carbons), db=number of double bonds.				

^*^Refers to comparisons to WT-FED sample group.

^†^To WT-FAS sample group.

^‡^To KO-FED sample group; Significances refer to ^2^*P*<0.001, to ^1^*P*<0.05.
